# Draft Genome Sequence of *Pseudomonas putida* Strain MTCC5279

**DOI:** 10.1128/genomeA.00560-13

**Published:** 2013-08-01

**Authors:** Vasvi Chaudhry, Mehar H. Asif, Sumit Bag, Ridhi Goel, Shrikant S. Mantri, Sunil K. Singh, Puneet S. Chauhan, Samir V. Sawant, Chandra Shekhar Nautiyal

**Affiliations:** Council of Scientific and Industrial Research–National Botanical Research Institute (CSIR-NBRI), Rana Pratap Marg, Lucknow, Uttar Pradesh, India

## Abstract

Here we report the genome sequence of a plant-growth-promoting rhizobacterium, *Pseudomonas putida* strain MTCC5279. The length of the draft genome sequence is approximately 5.2 Mb, with a GC content of 62.5%. The draft genome sequence reveals a number of genes whose products are possibly involved in plant growth promotion and abiotic stress tolerance.

## GENOME ANNOUNCEMENT

*Pseudomonas putida* strain MTCC5279 is a plant-growth-promoting rhizobacterium (PGPR) isolated from the roots of chick-pea (*Cicer arietinum* L. cv. Radhey) in a rain-fed area of Dholpur, Rajasthan, India. The strain *P. putida* MTCC5279 displayed plant growth promotional attributes such as the presence of auxin and the ability to solubilize phosphate and showed drought and salt tolerance (1).

Here we present the genome of *P. putida* MTCC5279 sequenced using a Roche Genome Sequencer FLX 454 system. A total of 237,769 reads were generated, covering a length of 78.2 Mb. These reads were assembled at an overlap size of 40 bp and 96% identity using the GS Assembler program, which yielded 193 contigs (longest contig, 152 kb; average contig, 27 kb). The consensus length of the draft genome sequence is 5.2 Mb, with ~14× coverage and a total GC content of 62.5%. The closest neighbor among its relatives was *P. putida* strain KT2440, with a genome size of about 6.18 Mb and GC content of 61.5% (2). Gene prediction and annotation were carried out using Glimmer3 (3), the Rapid Annotations using Subsystems Technology (RAST) server (4), the FindtRNA program (http://www.bioinformatics.org/findtrna/), RNAmmer (5), and the NCBI COG database (6). Metabolic pathways were examined through KAAS (the KEGG automatic annotation server) (7).

The draft genome contains 193 contigs, which were searched for the presence of open reading frames (ORFs) using Glimmer3. Of a total of 4,951 ORFs obtained, 4,692 ORFs were annotated against the NCBI-nr database, while 4,544 ORFs were annotated by use of the *P. putida* genomes (KT2440, GB1, BIRD, and F1). It was found that 148 genes were present in the NCBI-nr database but were absent in *P. putida* genomes. There were 299 novel predicted genes. The RAST server predicted a total of 4,900 coding sequences (CDS). Annotation covered 2,491 RAST subsystems (51%) with 1,176 CDS. Of these, 64 CDS were hypothetical proteins, while 1,588 (49%) did not belong to any subsystems and 871 of those corresponded to hypothetical proteins. In total, 143 tRNAs (67 in the forward strand and 76 in the reverse strand) and 5 rRNAs were identified using FindtRNA and RNAmmer, respectively. Around 4,544 genes were classified using clusters of orthologous groups (COG) analysis, resulting in 22 functional classes. The proteins associated with amino acid transport and metabolism were the most abundant group of COG (245 ORFs), followed by those associated with signal transduction mechanisms (244 ORFs) and transcription (185 ORFs). According to KEGG analysis, 5 pathways, *viz*., bisphenol degradation, carotenoid biosynthesis, polycyclic aromatic hydrocarbon degradation, lipoic acid metabolism, and ethylbenzene degradation, were found to be unique in *P. putida* MTCC5279 compared to other *P. putida* genomes (KT2440, W619, GB1, BIRD, and F1). The *P. putida* MTCC5279 genome presents several genes related to plant growth promotion and stress tolerance, including the *accD* gene encoding 1-aminocyclopropane-1-carboxylate deaminase and genes for the production of indole-3-acetic acid, siderophore, alginate, and the phosphatases. A more specific analysis of strain *P. putida* MTCC5279 will be reported in a future publication.

### Nucleotide sequence accession number. 

The genome sequence of *Pseudomonas putida* MTCC5279 is available in GenBank under the accession number AMZE00000000.

## References

[1] Srivastava S Chaudhry V Mishra A Chauhan PS Rehman A Yadav A Tuteja N Nautiyal CS 2012 Gene expression profiling through microarray analysis in Arabidopsis thaliana colonized by Pseudomonas putida MTCC5279, a plant growth promoting rhizobacterium. Plant Signal Behav. 7:235–245 22353860 10.4161/psb.18957 PMC3405686

[2] Nelson KE Weinel C Paulsen IT Dodson RJ Hilbert H Martins dos Santos VA Fouts DE Gill SR Pop M Holmes M Brinkac L Beanan M DeBoy RT Daugherty S Kolonay J Madupu R Nelson W White O Peterson J Khouri H Hance I Chris Lee P Holtzapple E Scanlan D Tran K Moazzez A Utterback T Rizzo M Lee K Kosack D Moestl D Wedler H Lauber J Stjepandic D Hoheisel J Straetz M Heim S Kiewitz C Eisen JA Timmis KN Düsterhöft A Tümmler B Fraser CM 2002 Complete genome sequence and comparative analysis of the metabolically versatile *Pseudomonas putida* KT2440. Environ. Microbiol. 4:799–808 12534463 10.1046/j.1462-2920.2002.00366.x

[3] Delcher AL Harmon D Kasif S White O Salzberg SL 1999 Improved microbial gene identification with GLIMMER. Nucleic Acids Res. 27:4636–4641 10556321 10.1093/nar/27.23.4636 PMC148753

[4] Aziz RK Bartels D Best AA DeJongh M Disz T Edwards RA Formsma K Gerdes S Glass EM Kubal M Meyer F Olsen GJ Olson R Osterman AL Overbeek RA McNeil LK Paarmann D Paczian T Parrello B Pusch GD Reich C Stevens R Vassieva O Vonstein V Wilke A Zagnitko O 2008 The RAST server: rapid annotations using subsystems technology. BMC Genomics 9:75. 10.1186/1471-2164-9-75 18261238 PMC2265698

[5] Lagesen K Hallin P Rødland EA Staerfeldt HH Rognes T Ussery DW 2007 RNAmmer: consistent and rapid annotation of ribosomal RNA genes. Nucleic Acids Res. 35:3100–3108 17452365 10.1093/nar/gkm160 PMC1888812

[6] Tatusov RL Natale DA Garkavtsev IV Tatusova TA Shankavaram UT Rao BS Kiryutin B Galperin MY Fedorova ND Koonin EV 2001 The COG database: new developments in phylogenetic classification of proteins from complete genomes. Nucleic Acids Res. 29:22–28 11125040 10.1093/nar/29.1.22 PMC29819

[7] Moriya Y Itoh M Okuda S Yoshizawa AC Kanehisa M 2007 The metabolic pathways were examined through KAAS (KEGG automatic annotation server): an automatic genome annotation and pathway reconstruction server. Nucleic Acids Res. 35:182–185 10.1093/nar/gkm321 PMC1933193 17526522

